# Corticosteroids for critically ill patients: an international survey of intensivists

**DOI:** 10.1186/cc10708

**Published:** 2012-03-20

**Authors:** F Lamontagne, N Adhikari, D Cook, KK Koo, F Lauzier, KE Burns, I Douglas, AF Turgeon, H Quiroz, Y Poulin, K Choong, N Ferguson, ME Kho, M Duffett, C Chant, O Lesur, MO Meade

**Affiliations:** 1 Université de Sherbrooke, Canada; 2 University of Toronto, Canada; 3 McMaster University, Hamilton, Canada; 4 University of Western Ontario, London, Canada; 5 Université Laval, Québec, Canada; 6 Denver Health Medical Center, Denver, CO, USA; 7 Johns Hopkins University, Baltimore, MD, USA

## Introduction

We surveyed intensivists to evaluate their stated use of systemic steroids in the ICU. The efficacy of steroids in septic shock and ARDS remains uncertain and clinicians' perceptions of competing indications and contraindications may jeopardize future randomized controlled trials (RCT). Knowledge of current practice will inform the design of future RCTs addressing the efficacy of systemic steroids in septic shock and ARDS.

## Methods

We designed and conducted a self-administered survey of intensivists practicing in academic settings with expertise in ARDS clinical research. We generated questionnaire items in focus group sessions with content experts and refined them through a standardized process of clinical sensibility, pilot and intra-rater reliability testing. Respondents used a four-point scale to grade how frequently they would administer systemic steroids in a 14 different clinical situations and reported their opinions of 16 near absolute indications or contraindications for systemic steroids. Local research staff distributed the survey to all intensivists practicing in the 11 centres (Canada and USA) with most patients enrolled in the OSCILLATE trial.

## Results

In total, 103 of 125 potential respondents returned completed surveys (response rate 82%). A majority of respondents 'almost always' prescribe systemic steroids in the setting of recent systemic steroid use and low blood pressure (93%), significant bronchospasm in a mechanically ventilated patient (93%) and vasopressor refractory septic shock (52%). A majority of respondents would 'almost never' prescribe steroids in severe community-acquired pneumonia (81%), ALI (76%) and ARDS (65%). One-half (50%) would 'almost never' prescribe steroids for severe ARDS (50%). The near absolute indications selected by a majority of respondents were 'known adrenal insufficiency' (99%) and 'suspicion of cryptogenic organizing pneumonia' (89%). The only near absolute contraindication selected by a majority of respondents was 'systemic fungal infection' (52%).

## Conclusion

Certain clinical conditions may prompt intensivists to almost always prescribe systemic steroids and reduce equipoise for future placebo-controlled trials. Moreover, this survey shows that in selected academic centres a majority of intensivists do not prescribe corticosteroids for pneumonia, ALI and ARDS.

